# Clinical efficacy of edaravone dexborneol in the treatment of acute ischemic stroke: meta-analysis

**DOI:** 10.3389/fneur.2025.1589307

**Published:** 2025-08-28

**Authors:** Haobo Gao, Hongtu Tan, Jiabin Wang, Dongyi Yang, Yangyang Liu, Tao Wu

**Affiliations:** Department of Intervention, The First Affiliated Hospital of Henan University of Chinese Medicine, Zhengzhou, China

**Keywords:** acute ischemic stroke, edaravone dexborneol, meta-analysis, clinical efficacy, safety

## Abstract

**Background:**

In China, edaravone dexborneol (ED) is often used to treat acute ischemic stroke (AIS), but internationally, ED is not commonly used. This study aimed to conduct a meta-analysis on the application of ED in AIS, so as to provide reference and guidance for clinical use in the future.

**Methods:**

Pubmed and Web of Science databases were searched for articles on ED in the treatment of AIS. Two researchers independently searched the articles based on pre-specified inclusion (randomized controlled trial, complete data, correct logic, English literature, etc.) and exclusion criteria (studies lacking objective reference standards, studies with follow-up success rates below 80%, etc.), and duplicate articles were excluded using Endnote. The search was set for the build time to December 2024. Then, the initially enrolled articles were subjected to information and data extraction and crosschecking, followed by meta analysis using the RevMan 5.3 software after screening. Primary outcome measures included clinical efficacy, safety, and NIHSS.

**Results:**

A total of 5 articles were included after screening, totaling 2,651 study participants. Subjects in the 5 articles were divided into an experimental group (ED treatment, *n* = 1,465) and a control group (other treatment regimen(s), *n* = 1,226). All the articles were of high quality and high reference value. According to Meta-analysis, no statistically significant difference was observed in the incidence of adverse reactions between the ED and control groups (95%CI = 0.67 ~ 1.04, *p* = 0.11), but the clinical efficacy in the experimental group exhibited significantly better outcomes compared to the control group (95%CI = 0.03 ~ 0.09, *p* = 0.0002). In addition, NIHSS (95%CI = −4.67 ~ −0.13, *p* = 0.04) and hs-CRP (95%CI = −1.90 ~ −0.23, *p* = 0.01) were lower in the experimental group than in the control group. Funnel plots were drawn for outcome measures with heterogeneity. The results showed that the funnel plot of the NIHSS scores and hs-CRP were basically symmetrical.

**Conclusion:**

While ED demonstrates short-term efficacy in improving neurological outcomes, its clinical applicability remains uncertain due to unresolved questions about drug diffusion and delivery in human brain tissue, which may fundamentally limit its long-term benefits.

## Introduction

Acute ischemic stroke (AIS) is a disorder of blood supply to brain tissues caused by various factors, which leads to ischemic and hypoxic necrosis and ultimately results in neurological dysfunction or even death (1). As the most common type of stroke, AIS accounts for about 69.6–70.8% of all strokes (2). According to the statistics of the World Health Organization, there are 1.5 million new cases of AIS globally each year, with an annual incidence rate of 116–219/100,000 (3). The incidence of AIS can reach more than 40% in patients over 60 years old with chronic diseases such as hypertension, diabetes mellitus and hyperlipidemia (4). AIS is characterized by sudden onset, rapid progression, and poor prognosis; approximately 30% of patients still suffer from irreversible neurological disorders despite active clinical treatment, which seriously affects their prognosis and quality of life (5).

Currently, there is still considerable controversy regarding the choice of therapeutic drugs for AIS, and there is a lack of optimal medication option (6). ED is a novel neuroprotective drug independently developed by China, which scavenges free radicals, protects brain cells, improves brain metabolism, and is mainly used in cerebral ischemic diseases (7). ED combines the pharmacological effects of both ED, achieving more significant results in inhibiting oxidative stress and inflammatory responses, which is expected to be a new choice for AIS treatment in the future. However, with the increasing number of clinical reports on the application of ED in recent years, the varied clinical effects greatly limit its clinical application.

Hence, the present study was to conduct a meta-analysis of clinical reports regarding ED in the treatment of AIS, so as to verify the potential of ED for future clinical application, and to provide new references and guidance for the treatment of AIS.

## Materials and methods

### Study protocol

The aim of this meta-analysis was to observe the clinical effects of ED in the treatment of AIS. This study has completed registration in PROSPERO and will be conducted in strict compliance with PRISMA guidelines.

### Inclusion and exclusion criteria

Inclusion criteria: (1) studies designed as a randomized controlled trial (RCT); (2) studies with subjects of patients with AIS; (3) studies adopting conventional treatment regimen + ED in the experimental group and conventional treatment regimen in the control group; (4) studies with complete data, where original data are available through the author(s); (5) English literature; (6) published after 2015; (7) Exclusion criteria: (1) studies with obvious logical errors; (2) studies with non-rigorous inclusion and exclusion criteria that may lead to biased results; (3) studies lacking objective reference standards; (4) studies with a follow-up success rate of less than 80%; (5) studies where authors have potential conflicts of interest; (6) studies lacking ethical approval statements, author informed consent statements, etc.

### Search strategy

Databases such as PubMed^1^ and Web of Science^2^ were searched for clinical studies on ED in the treatment of AIS from the date of establishment to December 2024 using medical subject heading combined entry terms. Keywords included edaravone, ED, randomized controlled trial, and acute ischemic stroke. Take Pubmed’s query box as an example: (((((((((edaravone dexborneol) AND (acute ischemic stroke)) AND (randomized controlled trial)) OR (Randomized)) OR (Double-Blind)) OR (Comparative Trial)) OR (cohort study)) OR (prospective)) NOT (rat)) NOT (mice).

### Literature screening and data extraction

Two researchers independently screened the articles and extracted the data. For any disputed articles, the inclusion and exclusion criteria were strictly followed, and experts were consulted to confirm the literature screening results. Data of interest were extracted from the articles using a self-made form, including: (1) basic information of the article: author(s), time, study title, journal, etc.; (2) subjects and methods: sample size, age, gender, time from onset to admission, blinding method, randomization; (3) intervention methods: treatment options; (4) outcome indicators: the clinical efficacy, incidence of adverse reactions, National Institute of Health stroke scale (NHISS) and hypersensitive C-reactive protein (hs-CRP) were recorded.

### Literature quality evaluation

The quality of the literature was assessed according to the risk of bias assessment criteria of the Cochrane system (8), including 6 domains of random allocation, clarity of the allocation scheme, blinding method, selective reporting, completeness of outcome data, and other bias. Each article was evaluated under each domain and categorized as low risk, high risk, or unclear. Grade A refers to articles meeting all the criteria above; grade B refers to articles meeting some of the criteria above; and grade C refers to articles meeting none of the criteria above. Risk of bias was independently assessed by two reviewers using the Cochrane RoB 2 tool. Discrepancies were resolved via consensus. Key domains with high/unclear risk included: Allocation concealment: 3/5 studies lacked explicit descriptions. Blinding of participants/outcome assessors: 4/5 studies were open-label, introducing performance bias. Selective reporting: 2/5 studies did not pre-register protocols, raising concerns about outcome reporting bias.

### Statistical analysis

RevMan 5.3 software was used for meta-analysis and graphical plotting. The relative risk (RR) was chosen as the effect indicator for enumeration data, and the weighted mean difference (WMD) for measurement data. *p* ≥ 0.10, *I*^2^ ≤ 50% suggested no heterogeneity among articles, and a fixed-effects model was used for analysis. *p* < 0.10, *I*^2^ > 50% suggested the presence of heterogeneity among articles, a random-effects model should be used for analysis, and funnel plots were drawn for verification (with basically bilateral symmetry indicating a low risk of bias). *p* < 0.05 was considered statistically significant.

## Results

### Literature screening results

**Figure 1:**
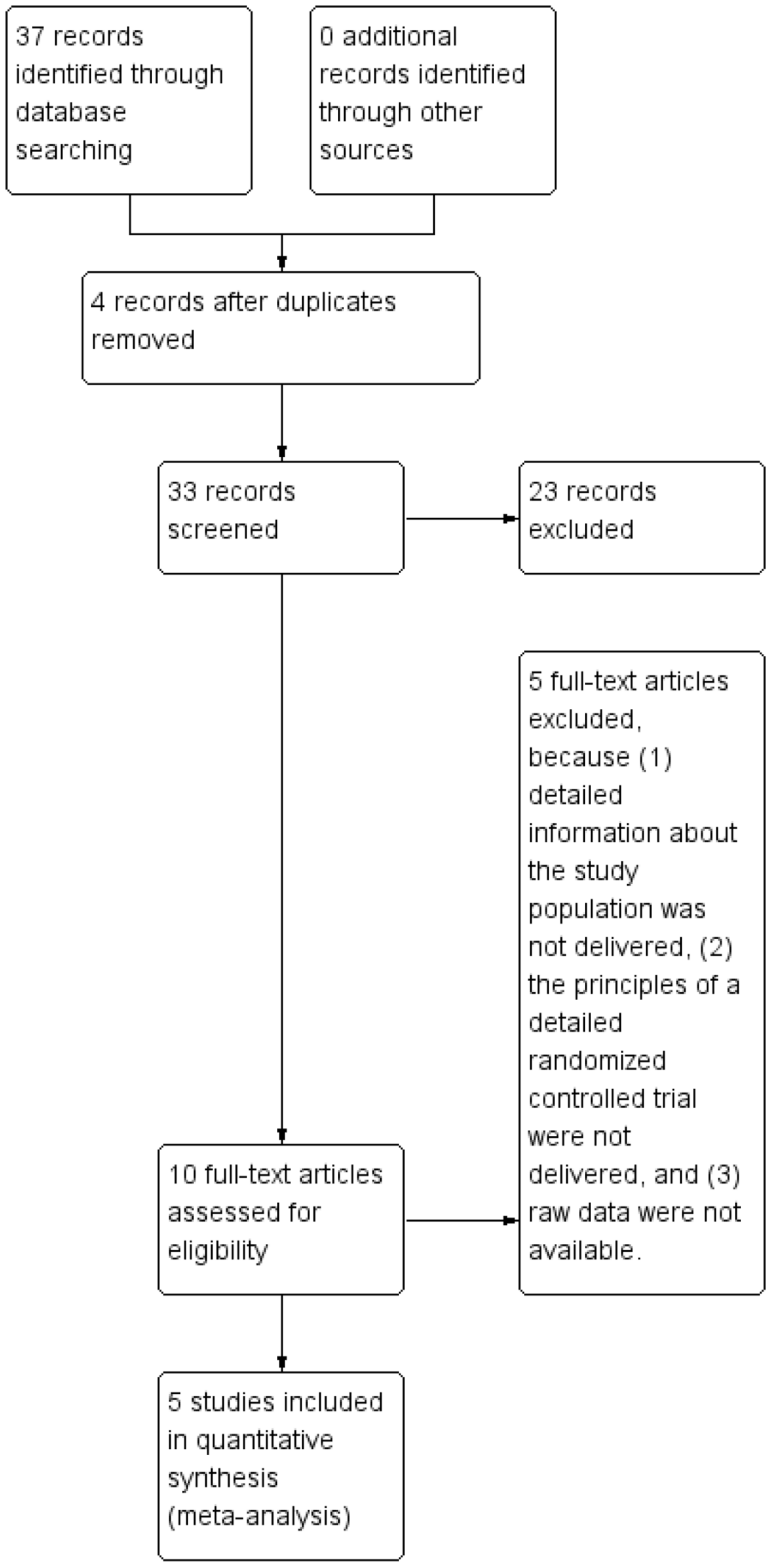
Screening process of literature.

**Figure 2:**
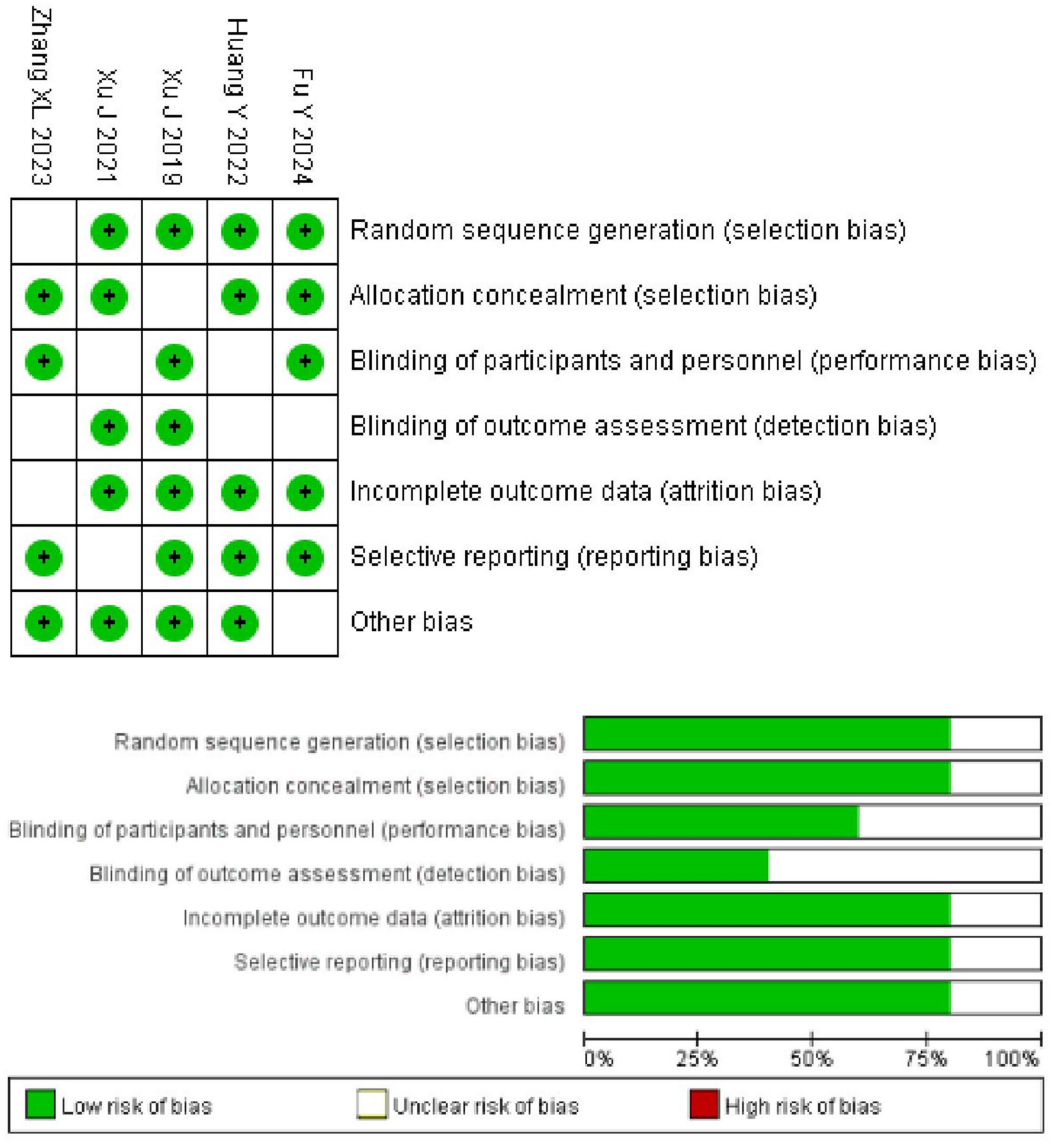
Risk assessment results from the literature.

A total of 37 articles relevant to ED in the treatment of AIS were searched from Pubmed and Web of Science databases, and 5 articles (9–13) were finally included for meta-analysis after duplicate removal by software, manual reading, and search criteria screening (the literature searching process is shown in Figure 1). The quality of the articles included in the study was primarily assessed as low to moderate risk, indicating a high reference value (Figure 2).

### Basic characteristics of subjects

The 5 articles enrolled involved a total of 2,651 subjects, among which 1,425 patients were treated with ED (the experimental group), and 1,226 patients were treated with conventional treatment (the control group). All these articles were assessed as grade B in terms of risks (Table 1).

**Table 1:** Basic characteristics of the literature.

Author/years	Experimental group			Control group				Risk level	Outcome indicators
	*n*	Age	Male/female	*n*	Age	Male/female	Treatment method		
Fu et al. (9), 2024	450	64.1 (56.0–69.8)	309/141	464	64.6 (57.1–71.4)	299/165	placebo-controlled study	B	(1)(2)(3)(4)
Huang et al. (10), 2022	15	62.8 ± 3.4	4/11	15	62.1 ± 2.6	5/10	Edaravone	B	(2)(3)
Xu et al. (11), 2019	289	59.11 ± 8.95	196/83	96	59.71 ± 8.33	65/29	Edaravone	B	(2)(3)(4)
Xu et al. (12), 2021	599	62.96 (55.38–68.96)	404/195	595	62.86 (55.72–71.12)	407/188	Edaravone	B	(2)(3)
Zhang et al. (13), 2023	72	-	-	56	-	-	Aspirin + Atorvastatin	B	(3)(4)

(1) Clinical efficacy, (2) incidence of adverse reactions, (3) NHISS, (4) hs-CRP.

### Clinical efficacy analysis of ED in the treatment of AIS

**Figure 3:**
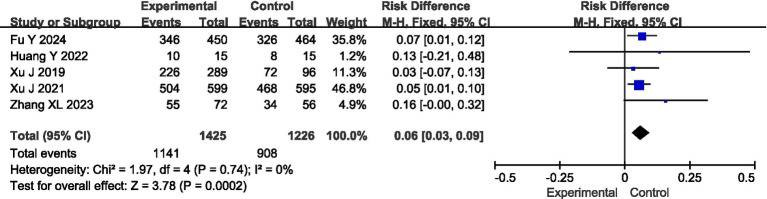
Meta-analysis of clinical efficacy.

All the articles reported the clinical efficacy of the patients, with no heterogeneity among them (*I*^2^ = 0%, *p* = 0.74), so a fixed-effect model was adopted for analysis. As indicated by the results, the overall response rate was higher in the experimental group compared to the control group (95%CI = 0.03 ~ 0.09, *p* = 0.0002) (Figure 3).

### Safety analysis of ED in the treatment of AIS

**Figure 4:**
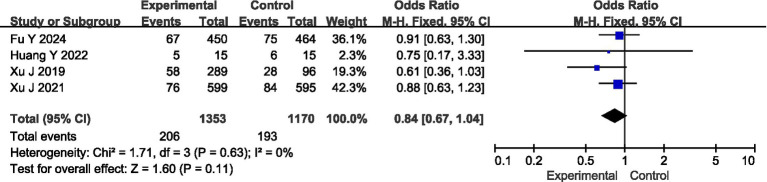
Meta-analysis of safety.

Four articles reported the safety of the patients, with no heterogeneity among them (*I*^2^ = 0%, *p* = 0.63), so a fixed-effect model was adopted for analysis. The analysis results showed that there was no statistically significant difference in the incidence of adverse reactions between the experimental group and the control group (95%CI = 0.67 ~ 1.04, *p* = 0.11) (Figure 4).

### NIHSS analysis of ED in the treatment of AIS

**Figure 5:**
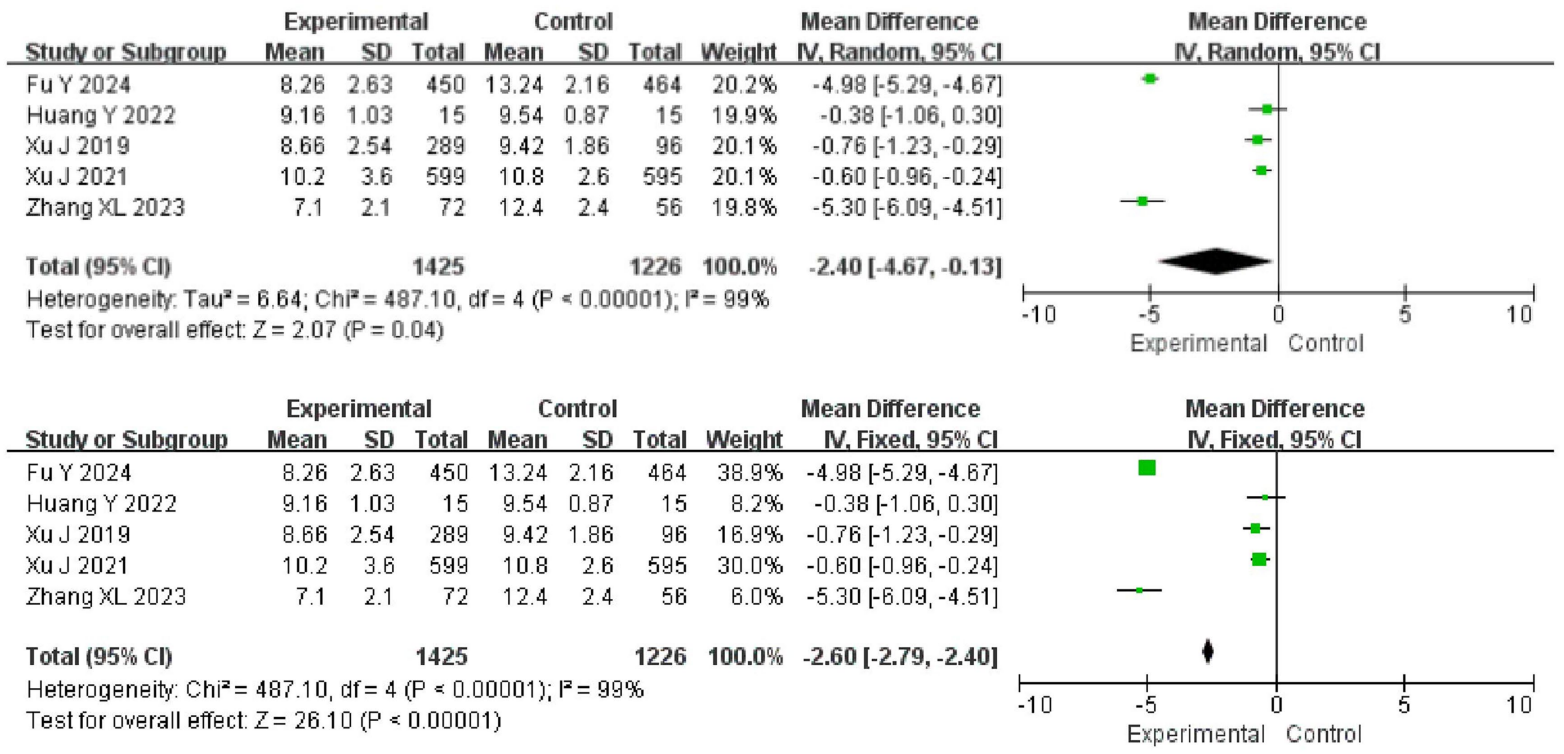
Meta-analysis of NIHSS scores.

Five articles reported the NIHSS scores of the patients, with certain heterogeneity among articles (*I*^2^ = 99%, *p* < 0.001), so a random-effects model was adopted for analysis. The results showed that the NIHSS score in the experimental group was lower than that in the control group (95%CI = −4.67 ~ −0.13, *p* = 0.04). After the analysis model was changed to a fixed-effect one, the difference between the two groups remained (95%CI = −6.09 ~ −0.451, *p* < 0.001) (Figure 5).

### Analysis of inflammatory response in AIS treated with ED

**Figure 6:**
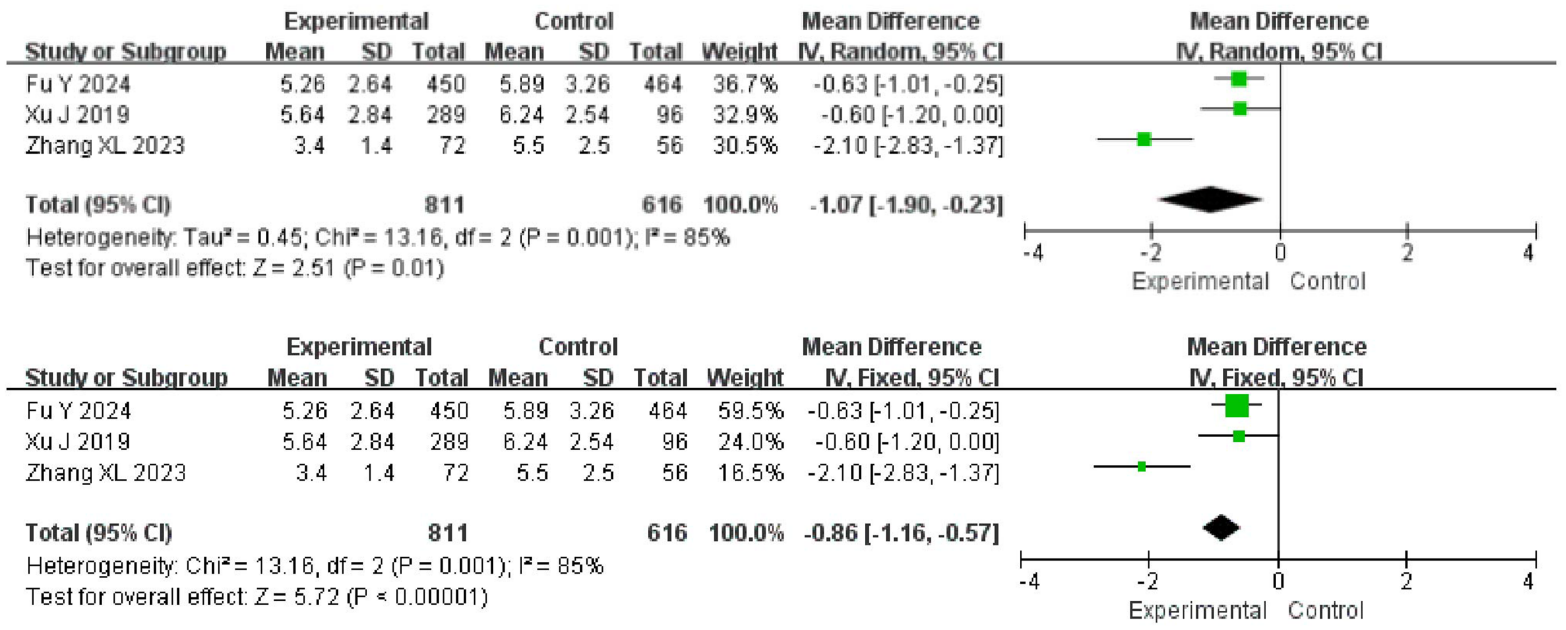
Meta-analysis of the inflammatory response.

Three papers reported hs-CRP in patients and there was heterogeneity among the papers (*I*^2^ = 85%, *p* = 0.001), which was analyzed using a random effects model. The results showed that hs-CRP was lower in the experimental group than in the control group (95%CI = −1.90 ~ −0.23, *p* = 0.01). After replacing the analytical model with a fixed-effects model, the difference between the two groups remained (95%CI = −1.16 ~ −0.57, *p* < 0.001) (Figure 6).

### Publication bias

**Figure 7:**
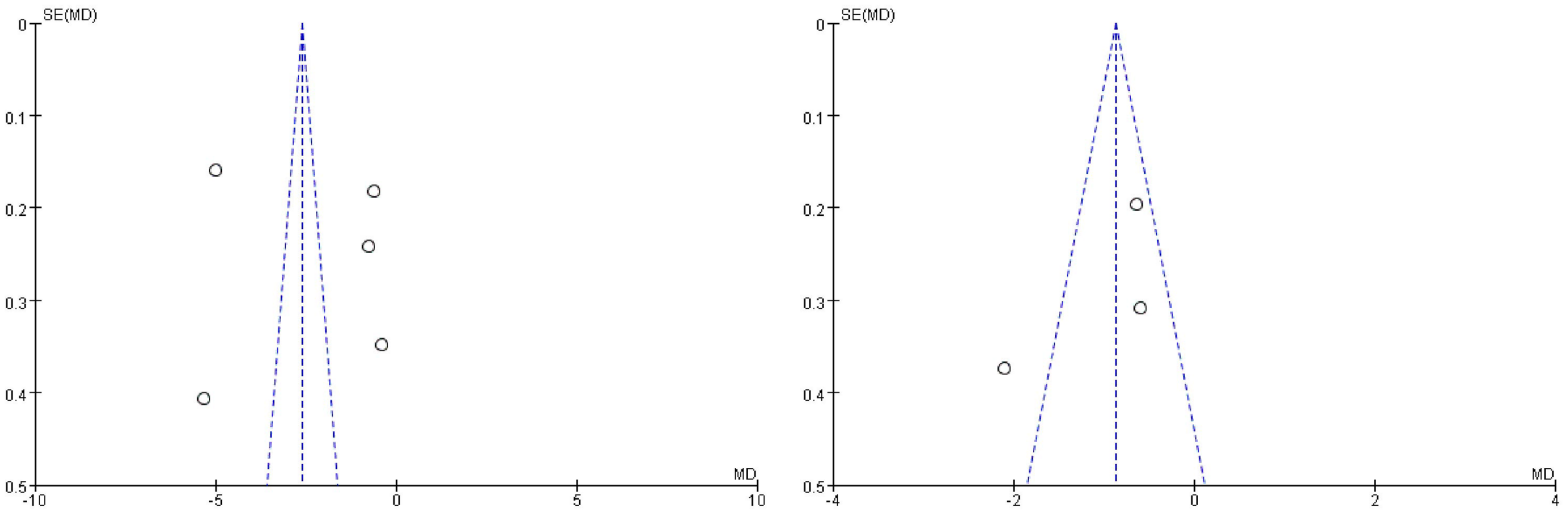
Funnel plotting indicators where heterogeneity exists.

Finally, funnel plots were drawn for outcome measures with heterogeneity. The results showed that the funnel plot of the NIHSS scores and hs-CRP were basically symmetrical, which proved that the publication bias was low, demonstrating its value for reference (Figure 7).

## Discussion

The primary findings indicate that ED treatment is associated with significantly improved clinical efficacy and reduced neurological impairment compared to standard therapy. Notably, ED demonstrated comparable safety profiles to controls, with no significant increase in adverse events. Additionally, ED-treated patients exhibited lower levels of systemic inflammation.

In recent years, as the global aging problem has become increasingly severe, the incidence of AIS has also shown an upward trend year by year, making it one of the major risk diseases that cause disability and death among patients, imposing heavy burdens on patients and their families (14). ED is a novel neuroprotective agent, and there is still a lack of authoritative guidance on its clinical application. In the present study, we systematically evaluated the therapeutic effects of ED on AIS, which provided a reliable reference for clinical practice. After screening, we identified 5 reports relevant to ED in the treatment of AIS, involving 2037 study cases, a relatively small number. This is attributed to the lack of widespread clinical application of ED at present. The moderate quality of included studies (all rated ‘B’ per Cochrane criteria) underscores the need for caution in interpreting pooled estimates. Open-label designs and incomplete outcome reporting may inflate treatment effects, particularly for subjective outcomes like ‘clinical efficacy’. High-quality, placebo-controlled trials with blinded assessments are essential to confirm ED’s true efficacy. However, studies on edaravone are not uncommon nowadays. For example, Zhao K et al. published a meta-analysis on edaravone for AIS in 2022 (15). Please note, however, that edaravone and ED are not the same drug; ED contains two active ingredients, edaravone and dexborneol, and is a compound based on edaravone, and is therefore not comparable to edaravone in actual clinical use. After assessment, all the identified articles showed a low risk of publication bias, indicating their high reference value. Through meta-analysis, we found that ED in the treatment of AIS resulted in higher clinical efficacy and better improvement in patients’ neurological functions, indicating its significant therapeutic potential in AIS treatment. Besides, there was no difference in safety between the experimental group and the control group, indicating the high safety profile of ED. Currently, it is clinically believed that elevated levels of oxygen free radicals and inflammatory factors in patients with AIS may lead to excessive adsorption of leukocytes by vascular endothelial cells, blockage of cerebral blood vessels, and ultimately ischemic injury (16). By reducing the levels of oxygen free radicals and inflammatory factors, ischemia–reperfusion injury will be mitigated and the prognosis of patients will be improved (17). Although thrombolytic therapy directly improves the oxidative stress response in the body, the invasive procedure may lead to further increases in the levels of oxygen free radicals and inflammatory factors (18). Ye et al. (19) confirmed that ED can act as a scavenger of oxygen free radicals and reduce the accumulation of reactive oxygen species in hypoxic–ischemic encephalopathy. Moreover, the addition of dexborneol further enhances the synergistic effects with edaravone, contributing to more significant anti-inflammatory effects and brain tissue-protective effects (20). The treatment effect in the experimental group is better than the control group in this study, as edaravone alleviates neuronal and endothelial cell injury in patients with AIS through mechanisms such as neurotoxicity inhibition, chronic inflammation, and neuronal protein expression regulation (21). For ischemic/reperfusion injury, dexborneol exhibits effective neuroprotective effects through various molecular pathways such as reactive oxygen species and nitric oxide synthase. In an *in vitro* study of ED by Chen et al. (22), ED alleviated subarachnoid hemorrhage and reduced neuronal apoptosis in rats in a short period of time, which also supports our view. In terms of safety, although no significant differences were observed between the experimental group and the control group herein, Hu et al. (23) still raised concerns about the drug safety of ED, as long-term use may lead to increased intracranial pressure, impaired coagulation function, and liver dysfunction. Therefore, more in-depth studies are needed to confirm the clinical dosage and duration of ED. While ED has demonstrated promising effects in the treatment of AIS, the specific mechanisms underlying its actions remain to be verified. Xu et al. (24) suggested that ED exerts its protective effect on cerebral ischemia/reperfusion injury by activating the Nrf2/HO-1 signaling pathway, which may preliminarily reveal the pathway of ED. Subsequently, we will perform further validation analyses to confirm the specific mechanism involved. Finally, by plotting funnel plots, we observed that the funnel plot for heterogeneous outcome measures (such as NIHSS) is basically symmetrically distributed, confirming a low risk of publication bias, which aligns with the results of the above-mentioned literature quality evaluation, indicating that the findings of this study have important clinical implications.

ED is thought to have a low molecular weight, which theoretically favors its diffusion in brain tissue. However, according to recent findings, even under optimal oxygen conditions, ED can only diffuse by a few millimeters (25). However, this diffusion barrier is not related to BBB integrity, but originates from the interaction between the physicochemical properties of the drug itself and the microenvironment of brain tissue. As noted in the study by Aksenov and Doubovikov (26), strategies to improve drug delivery in practical clinical applications are urgently needed—perhaps through local delivery systems, enhanced diffusion mechanisms, or new approaches such as nanoparticles, which will greatly improve the practical clinical application value of ED. Recently, a number of studies have attempted to break through this limitation through new delivery systems, such as the phosphorus tree-based macromolecular carrier developed by Ma et al. (27), which can co-deliver and fibronectin, and use the active permeability property of nanoparticles to deliver the drug to infarcted tissue. Xu et al. (24) confirmed that the permeability of blood–brain barrier could be indirectly enhanced by regulating Nrf2/HO-1 signaling pathway. These findings suggest that strategies relying solely on passive diffusion of small molecules may be difficult to meet clinical needs, and there is an urgent need to develop novel delivery technologies to overcome the tissue diffusion bottleneck.

There are some other limitations to this study. For instance, there may be some differences in the judgment of clinical efficacy among various articles, and the NIHSS score is also a subjective finding, both of which may introduce errors into the conclusions of the study. All included studies originated from China, limiting the generalizability of findings to other ethnic populations or healthcare systems. Geographical homogeneity may introduce biases related to variations in stroke subtypes, treatment protocols, and genetic factors. The absence of long-term follow-up data in our analysis represents a critical gap. While ED shows promise in reducing early neuroinflammation and oxidative stress, its effects on preventing post-stroke complications (e.g., recurrent stroke, cognitive decline) remain unknown. Another focal point to note is that two articles (9, 12) have disproportionately high weight (43.7 and 31.2%, respectively) due to large sample sizes, potentially skewing the pooled estimates. However, there are a limited number of studies on ED as a treatment of AIS, this issue cannot be addressed by including more studies. Further RCTs are required to verify the effect of ED on AIS. Future RCTs should prioritize extended follow-up periods and patient-centered outcomes. Due to resource constraints, this meta-analysis focused on PubMed and Web of Science. However, we acknowledge potential limitations in coverage and recommend future studies include additional databases such as Embase and the Cochrane Library. In this regard, we hope that the sample size may be increased in subsequent clinical studies, the randomization method be correctly adopted, the allocation concealment and blinding method be reasonably used, and relevant measures be objectively evaluated, to minimize the occurrence of various biases and to provide a more reasonable and accurate reference for clinical practice.

## Conclusion

Current evidence suggests that ED is safe and offers short-term improvements in the neurological function of AIS patients. In the future, ED may have significant clinical application as a treatment of AIS.

## Data Availability

The original contributions presented in the study are included in the article/supplementary material, further inquiries can be directed to the corresponding author.

## Footnotes

^1^ https://pubmed.ncbi.nlm.nih.gov/

^2^ https://www.webofscience.com
